# Molluscum contagiosum presenting as periorbital abscess in immunocompetent children

**DOI:** 10.1038/s41598-023-45320-y

**Published:** 2023-10-23

**Authors:** Moran Mor-Shaham, Joel Gutovitz, Oz Levinkron, Judit Krausz, Daniel Briscoe

**Affiliations:** 1 Present Address: Department of Ophthalmology, Emek Medical Center, Afula, Israel; 2 Present Address: Technion, Israel Institute of Technology, Haifa, Israel; 3 Department of Pathology, Emek Medical Center, Afula, Israel

**Keywords:** Paediatrics, Virology, Eye manifestations

## Abstract

Molluscum contagiosum presenting as a periorbital region abscess is unusual. The virus generally causes a self-limiting localized disease in children. Presentation as an abscess has been reported mainly in immunocompromised patients. We performed a retrospective study of ten children treated for Molluscum contagiosum infection presenting as periorbital abscess. Data investigated included age, immunocompetence, systemic antibiotic treatment, clinical findings, and histopathology. All children were immunocompetent. Bacterial cultures taken in six of the ten children were positive in two. Seven patients received oral antibiotics before presentation but required IV antibiotics on admission. One patient received IV antibiotics only. All antibiotic treatment had very limited effect. Two patients had no antibiotic treatment. CT imaging in one case where orbital cellulitis was suspected showed no significant intraorbital findings with anterior involvement only. Nine out of ten children had surgery and intra-operative cryotherapy at our center with immediate improvement and recovery. One child whose parents initially refused surgical excision had initial limited clinical improvement of periorbital swelling with antibiotics. However, the lesions were excised shortly following discharge from our hospital at another medical center with a complete cure. Molluscum is a cause of periorbital abscess in immunocompetent children which should be part of the differential diagnosis in periorbital/adnexal infection. Antibiotic treatment has a limited effect, and the abscess is most likely a virally triggered reaction. Surgical excision and intra-operative cryotherapy are curative of the disease in our experience.

## Introduction

Molluscum contagiosum is a common benign viral infection seen commonly in children. The disease is caused by a double stranded DNA virus of the Poxviridae family. Molluscum infects the human epidermis causing dome-shaped papules with an average diameter of 3–5 mm^1,2^. The infection is transmitted by contact with infected people or contaminated objects^1^. Several studies have shown a greater prevalence and higher number of lesions in patients with atopic dermatitis^3,4^ that may be explained by the immunologic changes and an impaired skin barrier in this disease. Molluscum is typically more severe with larger and more disseminated lesions in immunocompromised patients^1^.

In immunocompetent patients, lesions regress spontaneously over time in most cases^1–5^. Spontaneous regression of Molluscum lesions is generally accompanied by signs of inflammation including perilesional erythema^5^.

The diagnosis of Molluscum is based on physical examination with observation of the typical umbilicated papules. Dermatoscopy can aid diagnosis in some cases by identifying the punctiform, radial, or “mixed flower” patterns of the lesion vasculature^6^. The definitive diagnosis is made by biopsy and histopathology^2^.

Molluscum lesions may spontaneously resolve without treatment. While most cases resolve within six to nine months, some may persist for years^7^.

Periocular Molluscum lesions frequently affect the eyelid margins or the area near the palpebral aperture and may cause secondary unilateral follicular conjunctivitis or superficial punctate keratitis^8^.

Although periocular lesions may be self-limited with no need for intervention beyond conservative management, it is important to understand that the disease can also take an aggressive form. In some cases, eczematous reactions, pruritus, pain, discomfort, superinfection and hypersensitivity may appear^9^. Several reports and studies have suggested different therapies including topical, systemic and surgical interventions.

There are few reports of Molluscum presenting as periorbital abscess or eyelid cellulitis^10,11^. However, in one large series of 578 cases from all over the body including the head and neck, abscesses were present in about 11% of the patients^12^. It is not specified in that paper how many cases of abscesses were from the head and neck region or how many abscesses were in children. Abscesses are considered to be caused by discharge of Molluscum bodies to the dermis and the consequent release of proinflammatory cytokines with complement pathway activation. This is thought to induce a neutrophil reaction and abscess formation^13,14^. Our case series involves ten pediatric cases of aggressive Molluscum infection in immunocompetent children presenting as abscesses of the eyelid and anterior orbit. This would suggest that infections of the eyelids and periorbita caused by Molluscum could be more common than previously accepted in young children and clinical examination should always exclude it as a cause.

## Methods

We performed a retrospective review of ten pediatric patients suffering from eyelid Molluscum with abscess formation treated or examined at Emek Medical Center in Israel.

The study was permitted by the Emek Medical Center (EMC) ethics committee review board. Methods were carried out in accordance with relevant guidelines and regulations. Experimental protocols were approved by the EMC ethics committee review board. Waiver of informed consent was permitted by EMC ethics committee review board as the research was retrospective and anonymous, and information is not sensitive in nature presenting no threat to the rights and welfare of research subjects. Necessary informed consent was obtained from all parents or legal guardians for publication of identifying information and images.

Each patient presented with classical Molluscum lesions of the face and various parts of the body in addition to deep eyelid and orbital abscesses. Data including age, immunocompetence, treatment with antibiotics at the period of presentation, clinical findings and follow-up, histopathology, and bacterial culture results were reviewed.

All cases had a complete blood count and there was no leucopoenia or any irregularity implying immunodeficiency. All cases had normal development and weight gain. In Israel it is mandatory to check every woman during pregnancy for HIV. In addition, all newborns are screened by a mandatory blood test for SCID (Severe Combined Immunodeficiency). Every case in our cohort was screened negatively for any history of immunodeficiency, including the data discussed above, in addition to negative history of any symptoms, admissions or treatments connected to immunodeficiency. None of the children have systemic atopies. The cases were not screened for HIV as there was no medical indication.

## Results

**Figure 1:**
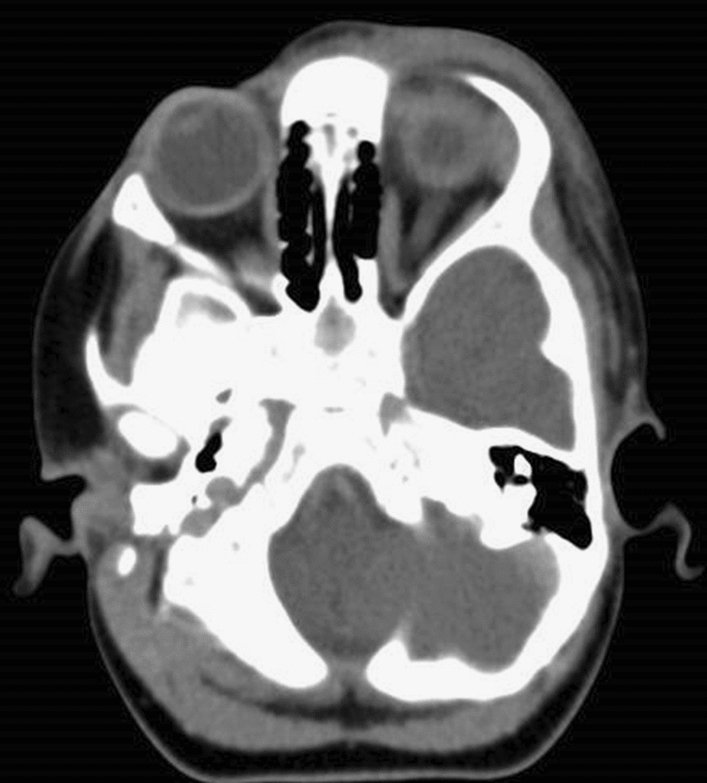
CT imaging of periorbital Molluscum abscess.

A total of ten cases of periorbital abscess and eyelid cellulitis caused by Molluscum contagiosum in immunocompetent children were included in our cohort (Table 1). Age ranged between 1 and 9 years old. There were seven boys and three girls, and all cases were admitted to hospital because of worsening signs and symptoms of infection despite antibiotic treatment given in eight of the ten cases. No similar adult cases presented, and this clinical picture was only seen in children. CT imaging was carried out in one out of ten cases, but there was no significant intraconal involvement of the abscess (Fig. 1).

**Table 1:** Summarizes all cases.

Case	Sex	Age	Oral antibiotic	IV antibiotic	Culture	Surgical excision and histopathology	Received imaging	Clinical outcome
1	Male	4	Yes	Yes	Negative	Molluscum	No	Full recovery
2	Male	1	Yes	Yes	Negative	Lesions excised at another center	No	Full recovery
3	Male	2	Yes	Yes	Staph	Molluscum	Yes	Full recovery
4	Male	2	Yes	Yes	Negative	Molluscum	No	Full recovery
5	Male	1	Yes	Yes	Negative	Molluscum	No	Full recovery
6	Female	1	Yes	Yes	Haemophilus	Molluscum	No	Full recovery
7	Female	9	No	No	None	Molluscum	No	Full recovery
8	Male	3	Yes	Yes	None	Molluscum	No	Full recovery
9	Female	3	No	No	None	Molluscum	No	Full recovery
10	Male	9	No	Yes	None	Molluscum	No	Full recovery

**Figure 2:**
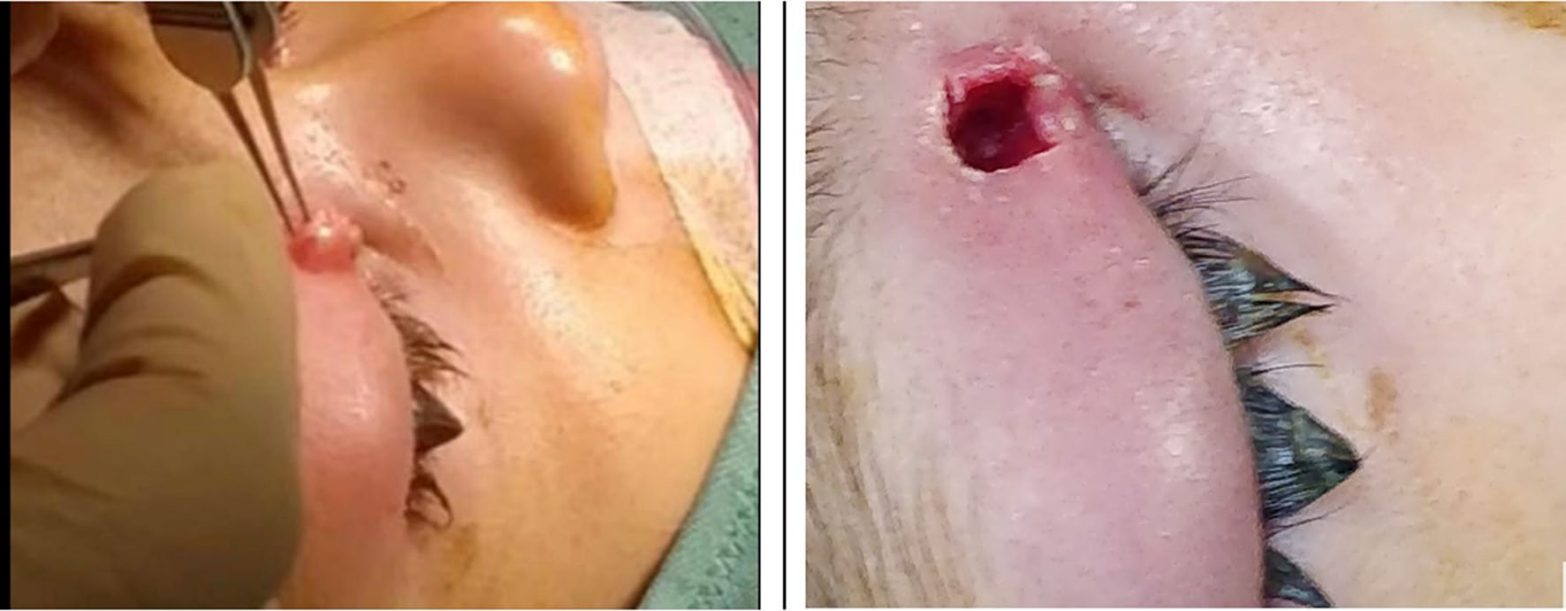
Typical surgical presentation of Molluscum periorbital cellulitis.

The children presented with large, expanding lesions and cellulitis of the eyelids and periorbita, which were concerning (Fig. 2).

There was localized pain and periorbital swelling with erythema, and in all cases, there were characteristic lesions on other parts of the body including the face, ears, trunk, arms, and genitalia. None of the children presented with restricted eye movements, severe orbitopathy, or clinical signs of serious disseminated infection such as fever, chills, nausea, vomiting, or diarrhea.

**Figure 3:**
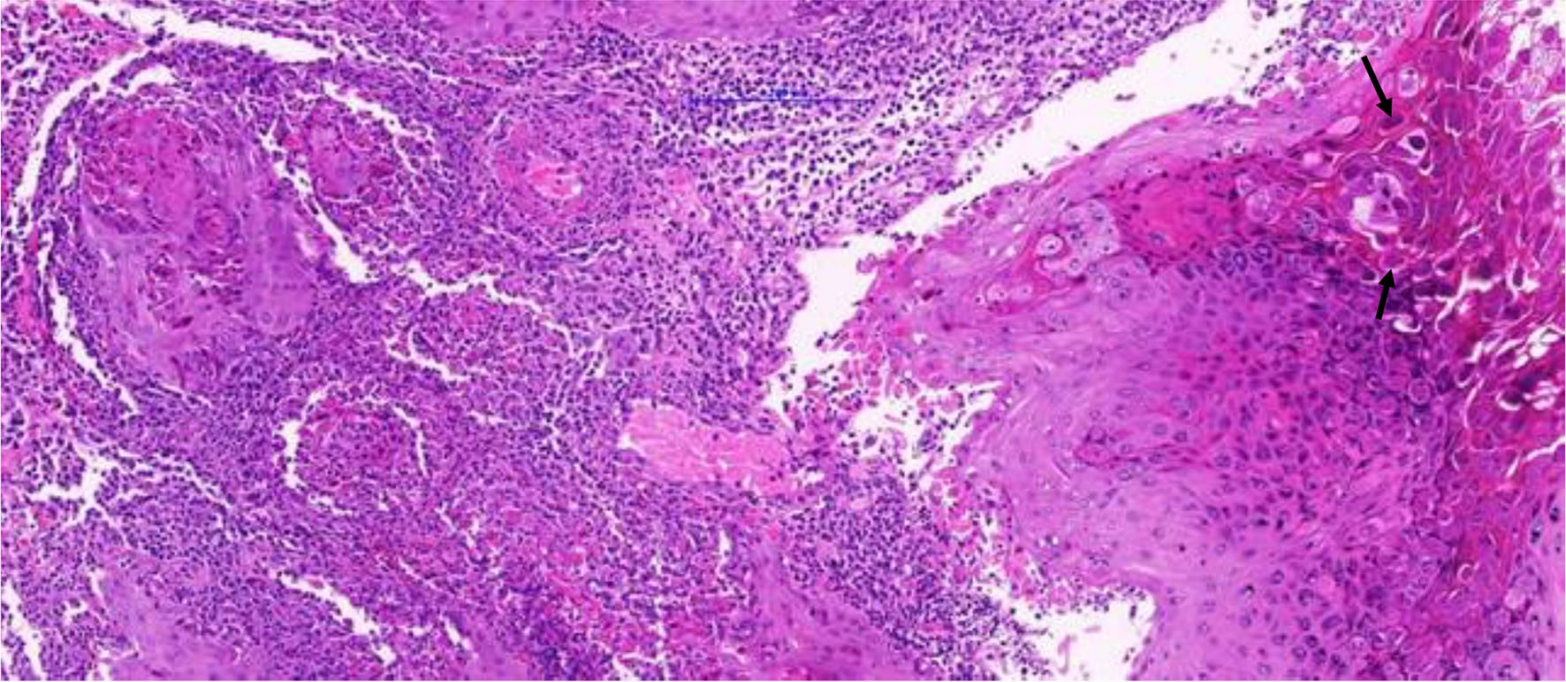
X15 Molluscum histopathology from periorbital surgical excision demonstrating granulation tissue with fragments of hyperplastic epithelium with many characteristic large Henderson–Patterson bodies (also called Molluscum bodies), two of which are indicated by the black arrows.

Blood analysis was normal in all but two of the cases, which showed lymphocytosis and neutrophilia. Although cultures were taken in six of the children, no bacteria were isolated in four of these cases. Two children had positive bacterial isolates (case 3 with Staphylococcus and case 6 with Heamophilus). Despite a negative bacterial culture, the child whose parents initially refused surgical excision of the Molluscum clinically improved with IV antibiotics, while the children with positive cultures had very limited clinical improvement with IV antibiotics. All ten children continued to surgical excision despite antibiotics. Histopathology demonstrated Molluscum in all nine of the ten patients who had surgery at our center, confirming the clinical diagnosis (Fig. 3). The tenth child underwent operation at another center and there is no histopathological confirmation of the clinical diagnosis.

**Figure 4:**
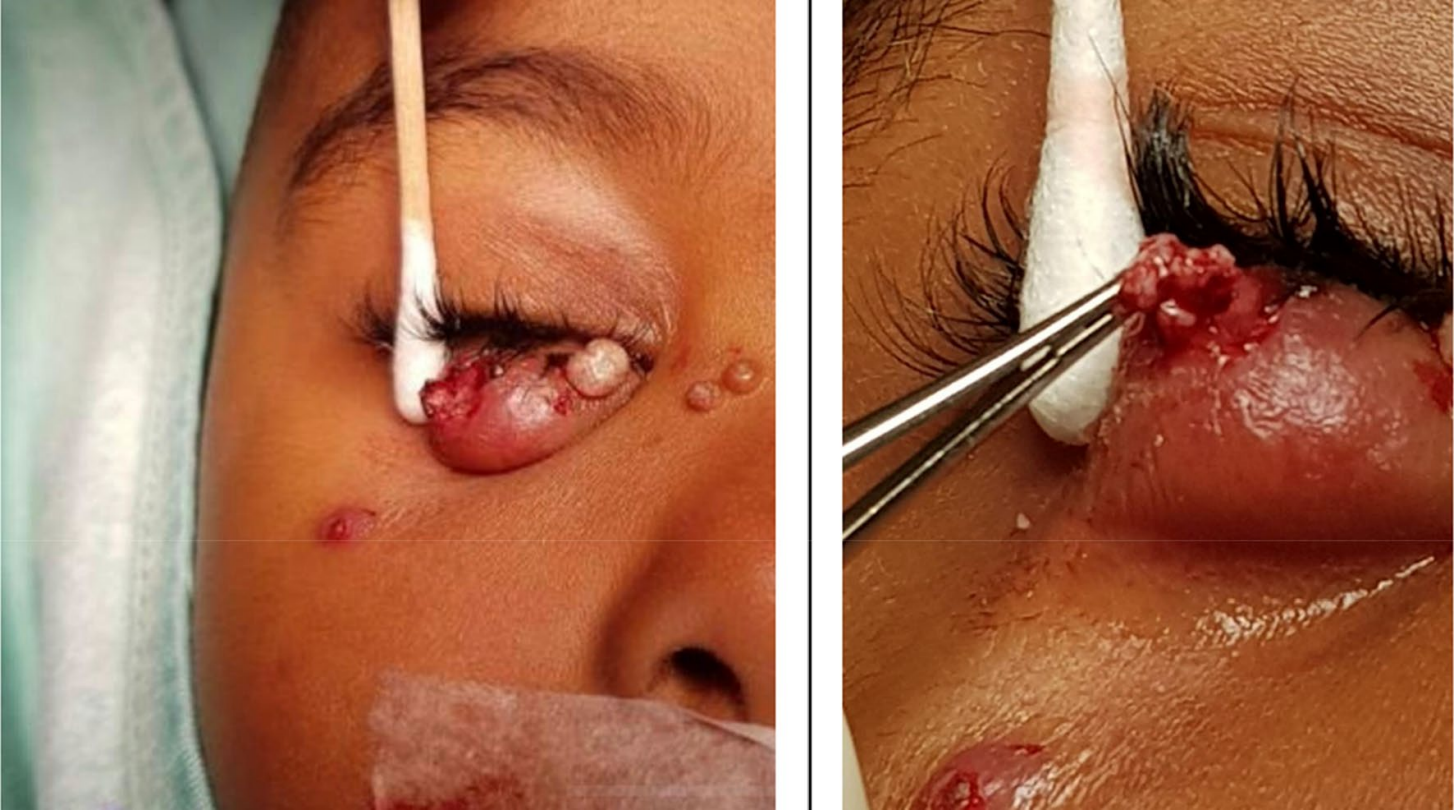
Typical surgical excision of a lesion.

Treatment with oral antibiotics or local antibiotic ointment was ineffective in all relevant cases. IV antibiotic therapy was ineffective in all but one case, where cellulitis was reduced in the eyelids, although the Molluscum lesion remained smoldering and were excised one week later at another facility. In all patients receiving antibiotic treatment, there was a limited effect only. Surgical excision of the viral lesions gave immediate resolution and cure (Fig. 4).

In all nine cases operated in our center, there was a substantial amount of characteristic white viral tissue, and surprisingly less pus than expected. The lesions invaded through the septum into the anterior orbital fat causing orbital edema and cellulitis.

All children recovered rapidly, and all signs and symptoms completely disappeared within a week of follow-up.

## Discussion

This case series of Molluscum periocular/adnexal abscess is distinctive in that it includes exclusively immunocompetent children only. There were no adult cases. Most cases in the literature of Molluscum with abscess formation are reported in children with immunodeficiency or HIV infection whereas all of our cases were immunocompetent^15–17^. Alrajeh et al. reported a case of an ulcerating eyelid Molluscum infection in an immunocompetent child that was surgically treated with complete cure^11^. We also had positive bacterial cultures in some cases as opposed to most of the literature, which describes positive bacterial culture mostly in immunocompromised patients. All children in our case series underwent surgery giving immediate improvement and cure of the disease. Published case series generally describe Molluscum in populations of all ages and include the whole body. One large case series described the presentation of abscesses in approximately 11% of Molluscum in their cohort, although they did not specify how many abscesses involved the head and neck or periocular region specifically^12^. Their cohort included patients of all ages. They estimated that only about 40% of Molluscum cases with abscess were clinically diagnosed. It would seem that the abscesses are virally induced. Molluscum with abscess was easily diagnosed clinically in all of our pediatric cases due to additional characteristic lesions elsewhere on the body. The abscesses can initially be misdiagnosed as bacterial abscesses when in fact they are actually virally induced reactive abscesses most probably caused by proinflammatory cytokines and complement pathway activation^13,14^. Some lesions may have had secondary bacterial infiltration from scratching, but this is unlikely to be the cause of the deep inflammatory infectious reaction observed. Broad-spectrum antibiotics had minimal effect on the abscesses in the cases where bacterial cultures were positive, and no cases had clinical systemic signs or symptoms. Surgical excision has an immediate curative effect, and although coverage with antibiotic may be indicated, its effect is limited at best in our experience.

Early periocular Molluscum literature described the disease in immunosuppressed patients, correlating indirectly with CD4 count. Molluscum, however, is most commonly seen in immunocompetent pediatric patients^1^. While aggressive Molluscum lid abscess is known to present in immunosuppressed patients^15–17^, its presentation in the immunocompetent pediatric population is typically less severe than in our cases^2^. It is possible, but unlikely, that the novel presentation of Molluscum with abscess, as in our cases, represents a new Molluscum virus variant. This hypothesis would need to be assessed by genetic testing in future presentations of the disease. Characteristic small, self-resolving papules are found in most immunocompetent children, as opposed to our case series, where Molluscum presented as painful, persistent, and aggressive periocular abscesses. Örnek et al. have already suggested the differential diagnosis of periocular infection in children should always include Molluscum, a diagnosis that can sometimes be evasive^18^.

Co-infection has been previously reported mainly in immunosuppressed patients^15–17^, although we think it is likely that the bacterial infection is mostly insignificant in immunocompetent cases such as ours. However, due to the gravity of complications that can occur with a periorbital abscess, it is very difficult not to give antibiotic cover to these children.

From our experience, it is likely that CT or MRI orbital imaging in immunocompetent children is unnecessary before surgery in cases of Molluscum abscess, as they do not penetrate into the intraconal fat space.

It has been well established that surgical removal or cryodestruction of the viral core are highly effective modes of treatment for Molluscum^9,18–20^. Periorbital Molluscium abscess has the potential to cause significant morbidity and therefore timely surgical intervention is probably the best mode of management even when antibiotic treatment has begun. We suggest that in these cases surgery should be scheduled as soon as possible.

## Data Availability

The dataset generated and analyzed during the current study is not publicly available, as we accessed patient information through the hospital database. The hospital database is not publicly available and is protected by privacy laws. Data can be made available from the corresponding author on reasonable request.
